# Mechanisms of Intramolecular Communication in a Hyperthermophilic Acylaminoacyl Peptidase: A Molecular Dynamics Investigation

**DOI:** 10.1371/journal.pone.0035686

**Published:** 2012-04-27

**Authors:** Elena Papaleo, Giulia Renzetti, Matteo Tiberti

**Affiliations:** Department of Biotechnology and Biosciences, University of Milano-Bicocca, Milan, Italy; King's College, London, United Kingdom

## Abstract

Protein dynamics and the underlying networks of intramolecular interactions and communicating residues within the three-dimensional (3D) structure are known to influence protein function and stability, as well as to modulate conformational changes and allostery. Acylaminoacyl peptidase (AAP) subfamily of enzymes belongs to a unique class of serine proteases, the prolyl oligopeptidase (POP) family, which has not been thoroughly investigated yet. POPs have a characteristic multidomain three-dimensional architecture with the active site at the interface of the C-terminal catalytic domain and a β-propeller domain, whose N-terminal region acts as a bridge to the hydrolase domain. In the present contribution, protein dynamics signatures of a hyperthermophilic acylaminoacyl peptidase (AAP) of the prolyl oligopeptidase (POP) family, as well as of a deletion variant and alanine mutants (I12A, V13A, V16A, L19A, I20A) are reported. In particular, we aimed at identifying crucial residues for long range communications to the catalytic site or promoting the conformational changes to switch from closed to open ApAAP conformations. Our investigation shows that the N-terminal α1-helix mediates structural intramolecular communication to the catalytic site, concurring to the maintenance of a proper functional architecture of the catalytic triad. Main determinants of the effects induced by α1-helix are a subset of hydrophobic residues (V16, L19 and I20). Moreover, a subset of residues characterized by relevant interaction networks or coupled motions have been identified, which are likely to modulate the conformational properties at the interdomain interface.

## Introduction

The current scenario on enzyme function and mechanisms reveals that conformational dynamics is indispensable for protein function, as well as that conformational transitions can involve mechanisms of conformational selection and induced fit, which can be considered as a special case in the catalytic network [1]. Several experimental evidences also support the role of conformational dynamics in catalysis [2], [3], even though it is still a matter of debate whether the enzyme dynamic motions contribute or not to decrease the chemical reaction barrier [1], [4], [5]. However, nowadays it is quite clear both from computational and experimental studies that enzyme conformational transitions are highly organized and correlated to enzyme specificity and efficiency. In particular the lowest frequency motions in protein dynamics are the most conserved at the superfamily and family level [6]–[11], as well as the more robust and less perturbed by silent mutations [9], [12].

Moreover, the recent advances in nuclear magnetic resonance (NMR) spectroscopy [7], [13], [14] and molecular simulations approaches [15], [16] make it possible to extract information on coupled motions and details on protein dynamics and allostery, thanks for example to the analysis of the cross-correlations of atomic fluctuations [17]–[20] or long range pathway of communicating residues [21]–[23]. These techniques allow to trace “communication paths” between distal residues in the protein structure and to define how a root residue can regulate or influence other distal residues [23]. In general, residues with patterns of clearly correlated movements are known to be associated with protein thermal stability and functional roles [19], [24], . Moreover, it has been suggested that critical residues associated either with protein function or with the maintenance of the three-dimensional (3D) architecture generally coevolve [26], [27]. It has also been demonstrated by NMR investigation that a tight connection exists between protein motion timescales, implying that the motions on picoseconds (ps) or nanoseconds (ns) timescale provide information on events likely to happen on larger timescale and modulating the most important conformational transitions [3]. Therefore, the description of networks of weak intramolecular interactions and of “communicating” residues within the structure, in a dynamic perspective, can provide relevant information on the mechanistic aspects related to a protein system.

In this context, we focus our attention on protein dynamics signatures of acylaminoacyl peptidase (AAP), which belong to the prolyl oligopeptidase (POP) family [28]. Acylpeptide hydrolases catalyze the removal of an N-acylated amino acid from blocked peptides of various size and with different acyl groups at the N-terminus [29], [30]. Only one X-ray structure of a member of the AAP subfamily, the acylaminoacyl peptidase from *Aeropyrum pernix* K1 (ApAAP, E.C. 3.4.19.1) [31] is presently available, and only a few structural and short dynamics studies have been provided so far [31]–[35], none of them with the aim of studying long range structural transmission. ApAAP is an esterase [36] and is characterized by a hyperthermophilic character [32], [37]. Esterases are among the most widely used classes of enzymes in several industrial processes, including stereospecific hydrolysis, transesterification, ester synthesis and other organic biosynthesis reactions. AAP, along with other members of the POP family [28], [38], [39] is also a target of pharmacology interest. In fact, a deficiency in human AAP was correlated to the development of cancer diseases [40], as well as it has been demonstrated that AAP inhibition favors apoptosis [41]. AAP is also a sensitive target for organ-phosphorus compounds, thus being a potential site for cognition-enhancing drugs [42].

**Figure 1:**
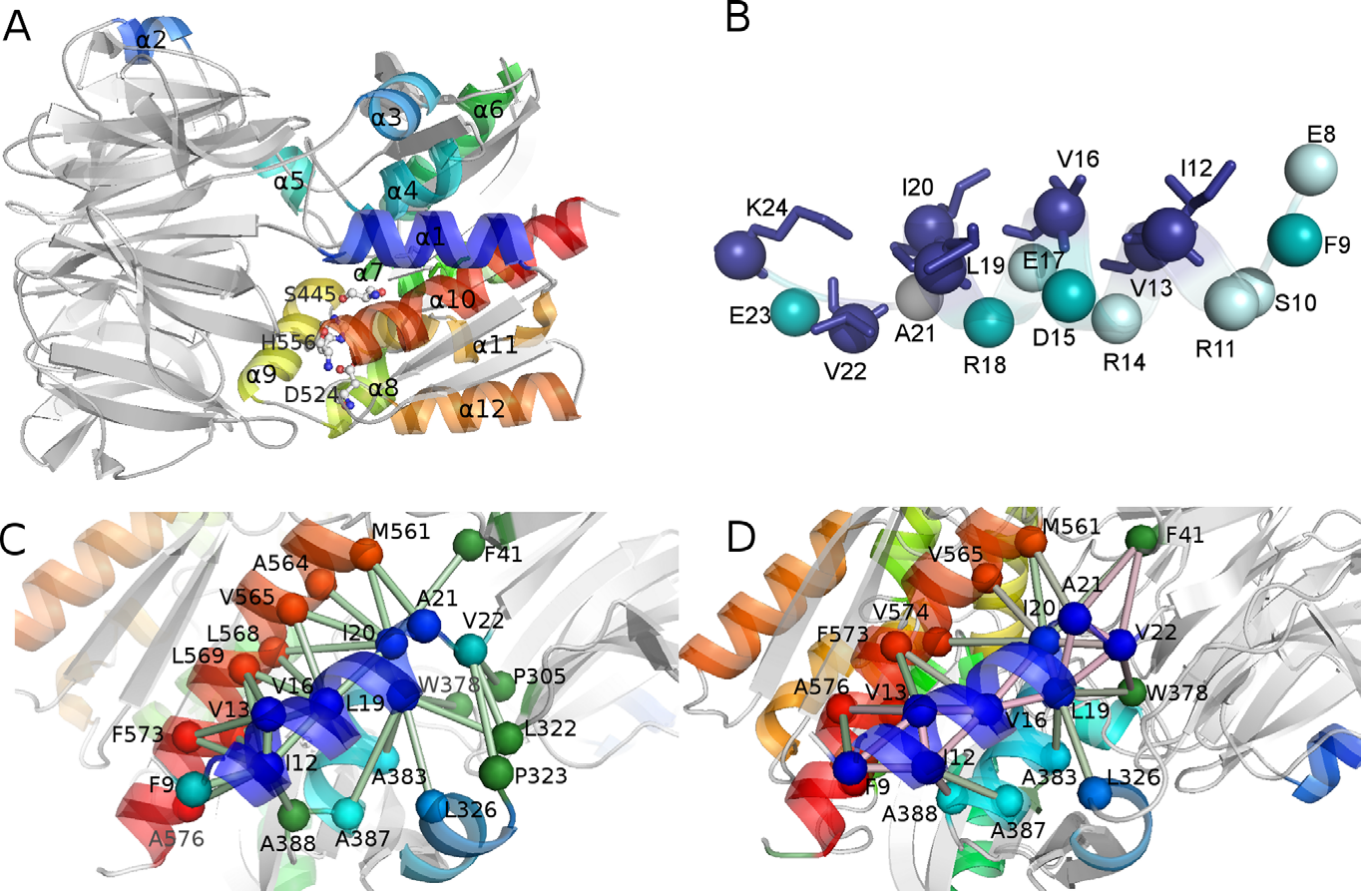
ApAAP 3D structure, *in silico* alanine scanning and hydrophobic interaction networks. A) The secondary structure elements of ApAAP and its 3D architecture are shown, with α-helices colored in different shade of colors from the N- (blue) to the C-terminal (red) extremity. The catalytic triad (S445, D524, H556) is shown as sticks and spheres. B) The residues which have been predicted to destabilize (blue; I12, V13, V16, L19, I20, V22 and K24), partially destabilize (cyan; F9, R18, D15, E23) or not influencing (pale cyan; E8, S10, R11, R14, and E17) the ApAAP 3D structure, upon *in silico* alanine mutations, by a consensus of three different programs (see Materials and Methods) are shown as spheres. Side chains of residues which, upon *in silico* alanine mutations, are predicted to have the most detrimental effects on protein stability (blue) are shown as sticks. Individual **ΔΔ**G values calculated by FoldX, I-Mutant and PoPMusic are reported I Table S1. C–D) The network of intramolecular hydrophobic interactions involving α1-helix residues in the X-ray structure (C) and as derived by the molecular dynamics (D) are shown as spheres connected by sticks.

The 3D structure of ApAAP is a symmetric homodimer with each subunit composed of two different domains, i.e. a β-propeller domain (residues 1–324) and a C-terminal α/β hydrolase domain (residues 325–581) (Figure 1A) [31]. ApAAP domain organization strictly resembles other members of the POP family from eukaryotes and prokaryotes [28]. The catalytic triad is located in the C-terminal hydrolase domain (Ser445, Asp524 and His556, Figure 1A). Particular attention was devoted in the literature to the role of the N-terminal α-helix (α1, residues 8–21 in ApAAP, pdb entry 1VE6 [32], Figure 1A) which is a common structural feature of prolyl oligopeptidase (POP) family [28], even if it is not particularly conserved in the primary sequence along the POP family [32]. The N-terminal α-helix connects the β-propeller domain and the catalytic domain [36], as well as it provides, along with elements located in the C-terminal domain, part of the dimerization interface in the ApAAP dimer [31]. In fact, it has been shown that deletion of the whole N-terminal α1-helix (deletion of residues 1–21, Δ21-ApAAP) affects temperature-dependence of ApAAP activity [32]. However, the 3D structure of Δ21-ApAAP has been experimentally solved (pdb entry 2QZP) and does not show modification in the overall structure and the dimerization properties [36]. It is also known that the effects of α1-helix on thermal stability are not ascribable to its charged residues [32]. These evidences, along with the current view on decoupling between thermal inactivation and thermal unfolding in different enzymes [43], can stimulate further investigation in a dynamic framework to depict not only the intramolecular interactions exploited by the α1-helix but also the dynamical communicating residues which mediate long range effects.

In light of the above observations, the present contribution, by *in silico* alanine scanning, hundreds nanoseconds all-atom molecular dynamics (MD) simulations of wild type, deleted and mutant ApAAP variants (Table 1), provides a description of coupled motions and networks of intramolecular interactions and their dynamical communications, with particular attention to the N-terminal α1-helix and other interdomain interface regions.

**Table 1:** Summary of the multi-replica all-atom MD simulations.

Protein system	Duration per *replica*	Starting structure
Wt ApAAP	100 ns×2	X-ray structure of wt ApAAP in its closed conformation (pdb entry 1VE6)
Wt ApAAP open	120 ns	X-ray structure of wt ApAAP in its open conformation (pdb entry 3O4G)
ApAAP-Δ21	100 ns	X-ray structure of ApAAP-Δ21 (pdb entry 2QZP)
ApAAP-I12A	100 ns	In silico mutation starting from the X-ray structure of wt ApAAP
ApAAP-V13A	100 ns	In silico mutation starting from the X-ray structure of wt ApAAP
ApAAP-V16A	100 ns	In silico mutation starting from the X-ray structure of wt ApAAP
ApAAP-L19A	100 ns	In silico mutation starting from the X-ray structure of wt ApAAP
ApAAP-I20A	100 ns	In silico mutation starting from the X-ray structure of wt ApAAP

## Results

### *In silico* alanine Scanning and Networks of Hydrophobic Interactions of the N-terminal α1-helix Residues Support a Role for Hydrophobic Interactions in Mediating Intramolecular Communication and Protein Stability

α1-helix has been demonstrated crucial for ApAAP structure and stability, even if the mutations of its charged residues alone did not mimic the deletion of the whole α1-helix, as demonstrated by Feng’s group [32], suggesting that the determinants of the effects transmitted by the α1-helix has to be found in other interactions that the helix can exploit.

As a preliminary step, before molecular dynamics (MD) simulations, we used a consensus of three different methods for *in silico* alanine scanning to have an estimate of the putative hotspots for protein stability in α1-helix. We select the methods characterized by high prediction performances of the effects of single mutations on protein stability [44] (see Materials and Methods). Most of the alanine mutations in ApAAP are predicted to have partial or significant destabilizing effects, with the most relevant effects related to hydrophobic residues I12, V13, V16, L19, I20 and V22- (Figure 1B, Table S1). The calculations suggest a role for hydrophobic interactions in modulating the effects of the α1-helix, and they also provide a selection of residues for further investigations.

Interestingly, the effects transmitted by the α1-helix on the protein structure have not been described in details yet from a structural and dynamics perspective. In fact, the deletion of α1-helix seems not to affect protein dimerization [36], therefore indicating a role in intra-monomer stabilization and structural intramolecular communication. A minor role of α1-helix in dimerization is also suggested by the analysis of the interactions between the two ApAAP monomers in the dimeric structure (Table S2). Interestingly, it was recently shown that the conformation of one monomer within the ApAAP dimer is independent of the conformation of the other, so that the two monomers act independently and that the dimerization concurs to structure stabilization [33]. In light of these observations, and considering the complexity of the large multi-domain protein under investigation, we decided to focus our attention on the dynamic properties of the monomeric form.

We monitored, in the MD ensemble, the persistence of the hydrophobic interactions, which were previously observed in the ApAAP X-ray structure, for each of the α1 residues, along with the surrounding of each of the α1 hydrophobic residues in the MD ensemble. This allow to specifically identify hydrophobic interactions formed during the dynamics around the native state (Table S3 and Figure 1C–D). It turns out a network of interactions which from the α1-helix brings to the C-terminal helix (α13), α4 and α5 and the β-strands in the inner part of the β-propeller domain. The most connected α1 residues in the native dynamics are I12, V16, L19 and I20, with V16 also acting as a mediator of interactions within the α1-helix itself (Figure 1D). V13 seems to play a less relevant role. Some interactions present in the static X-ray structure are lost or feature a persistence below a significant cutoff, with particular regard to interactions with residues 322, 323 (close to α3), 565 and 568 (α13) that are replaced by other interactions in the same regions. Moreover, new hydrophobic interactions appear during MD simulations, with the effect to enforce the intra-helical networks in α1 and interactions with the β-propeller and the helices α4, α5 and α13 (Table S3 and Figure 1C–D).

### α1 Influences Long Range Protein Flexibility Pattern in ApAAP

No detailed studies aimed at revealing overall protein dynamics, as well as distal effects and communication between residues in the structure have been carried out on AAP, or more in general on POP fold, as far as we know. Considering the relevance of this protein fold and its complex multi-domain structure, a description of the dynamics fingerprint can be very informative for the rationalization of the available data in the literature and for future experimental investigations. Therefore, all-atom explicit solvent MD simulations have been carried out at 300 K for the wild type ApAAP in its closed and open conformations. Moreover, MD simulations were also carried out for an ApAAP variant bearing a deletion of α1 residues (ApAAP-Δ21), as well as I12A, V13A, V16A, L19A, I20A ApAAP mutants (Table 1), which were suggested as crucial elements of α1-helix in the *in silico* alanine scanning (Figure 1B).

ApAAP-Δ21 simulation does not show, along the simulation time, significant modifications of the secondary structural elements with respect to the wild type counterpart. In order to better evaluate differences in flexibility between ApAAP-Δ21 and wild type ApAAP, the rmsf profiles were calculated both as average profiles on 10 ns time windows and rmsf profiles from the whole simulations. In particular, rmsf profiles of wt ApAAP and ApAAP-Δ21 have been compared both from the MD ensemble and upon application of the Principal Component Analysis (PCA) to the MD trajectories. There is a general good agreement (with correlation coefficients >0.75) between the raw rmsf profiles and the ones derived by filtering the MD trajectories on the first 3 principal components or on the essential subspace that accounts for the 70% of the total variance.

**Figure 2:**
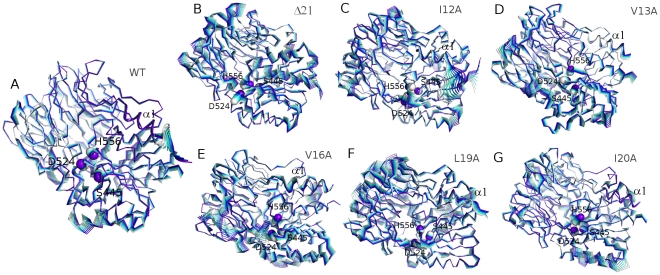
Protein dynamics fingerprint for wt, Δ21, and mutants ApAAP variants. The projections of the displacement described by the first principal component on the 3D structure are shown for wt (A), Δ21 (B), I12A (C), V13A (D), V16A (E), L19A (F), and I20A (G) ApAAP variants with the different simulation frames colored with different shade of colors from light cyan to purple. The catalytic triad and the α1-helix are shown as spheres and cartoon, respectively. The analyses were also carried out for the second and third components, which provide the same general view and are therefore not presented here.

ApAAP-Δ21, in agreement with suggestions from the X-ray B-factors [36] shows a higher flexibility scattered in several region of the 3D structure belonging to both the N-terminal and the C-terminal domains, suggesting the involvement of long range transmitted effects (Figure 2A). To better discriminate the effects induced by the α1- helix deletion on the rest of the protein structure, the projections of the displacement described by the first 3 principal components on the simulated frames have been analyzed (Figure 2) in both the wt (Figure 2A) and ApAAP-Δ21 (Figure 2B) variants. The 3 first principal components collectively account for more than 40% of the total motion and are therefore a suitable subspace to analyze protein dynamics. The deletion of the N-terminal α1-helix causes both local effects on its surrounding helices in the C-terminal domain, as well as long range perturbations both on the upper part of the β-barrel domain and on the catalytic site, affecting in particular the catalytic residues D524 and H556 (Figure 2B).

To gain further insights about the individual contribution of each α1 hydrophobic residue to the effects induced on protein flexibility through the structure, PCA was also carried out on the MD ensemble for I12A, V13A, V16A, L19A, I20A ApAAP mutants. V13A mutation (Figure 2D) does not significantly affect the principal motions and it is almost close to the wt dynamic fingerprint, whereas the other mutations have well-defined dynamics effects which mimic the deletion of the whole α1-helix (Figure 2). In particular, I12A mutation induces slight long range effects only in specific and well-localized regions. In the catalytic site, I12A mutation affects only D524 (Figure 2C). I20A mutation alters the ApAAP native dynamics mostly for the catalytic H556 (Figure 2G). L19A mutation is the alanine mutation here investigated that more effectively established a dynamic Δα1-like fingerprint in the β-barrel domain and in the surrounding of the α1-helix, and to a small extent in the catalytic site (Figure 2F). At last, V16A is the only alanine mutation which acts both long range and alters the dynamic patterns of the catalytic residues H556 and D524 (Figure 2D), as it was previously shown for the α1 deletion (Figure 2B).

### Salt Bridge Networks and Networks of Dynamical Coupled Residues in ApAAP MD Ensemble

The aforementioned analyses on intrinsic protein flexibility provide only a general view about the protein regions perturbed by the α1-helix deletion, and not the molecular details and a punctual identification of changes in the intramolecular interaction networks. However, the MD framework here provided allows also to evaluate if the lack of α1-helix causes perturbation in the intramolecular interactions. Moreover, if the appropriate tools are employed, it is also possible to define the “channels” of structural communication between protein residues in the MD ensemble, as it will be discussed in this section. These analyses disclose not only the effect induced by the N-terminal α-helix deletion, but they can also provide a detailed and general description of relevant interactions and coupled motions between the residues in native ApAAP dynamics.

#### The most populated clusters of salt bridges concur in the maintenance of the architecture of the catalytic site and to connect it to distal regions

The protein structural stability generally results from a delicate balance between different weak intramolecular interactions. Electrostatic interactions and in particular charge-charge interactions, have been shown to play a crucial role for protein stability [45]–[47], featuring both local and distal extremely variable effects related both to their attractive or repulsive nature. Nevertheless, charge-charge interactions have also shown to be highly flexible and cooperatively organized in networks in the protein structure [46], [48]. Therefore they are a suitable subset of intramolecular interactions that can be monitored in the MD ensemble to define perturbations induced by mutations, as we recently showed for a battery of mesophilic-like mutants of a cold-adapted enzyme [49].

In light of the above scenario, we investigated in details the persistence of salt bridge interactions as well as their organization in clusters of interconnected or spatially closed charged residues in the MD ensemble of the wild type ApAAP.

**Figure 3:**
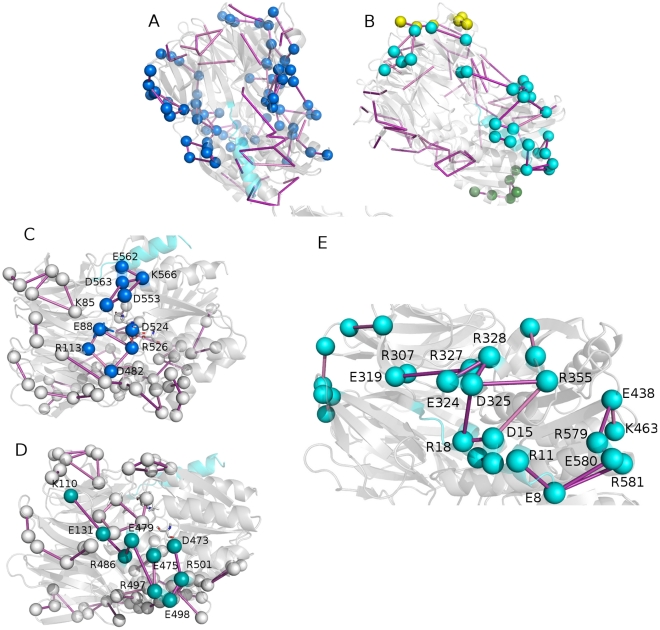
Salt bridge clusters in wild type ApAAP. Salt bridges belonging to cluster 1 (A, blue), cluster 2 (B, E, cyan) and clusters 3 (B, yellow) and 4 (B, green) are shown as spheres and connected by sticks. C–D) Details on salt bridges belonging to cluster 1 and located in proximity of the catalytic site. E) Details of some salt bridge networks located in cluster 2. The α1-helix is highlighted as cyan cartoon. The sticks connecting the salt bridges are colored according to the persistence of the interactions in the simulations (from light to dark magenta for increasing persistence values).

In agreement with its hyperthermophilic character, ApAAP shows few highly populated and well-interconnected main clusters of salt bridge interactions (Figure 3, Table S4). In fact, 4 main clusters are identified, populated by 69, 27, and 6 residues, respectively (Figure 3A–B, in blue (cluster 1), cyan (cluster 2), yellow (cluster 3) and green (cluster 4), respectively).

Cluster 1 includes residues which are distributed over the external faces of ApAAP structure, as well as several interdomain interactions (Figure 3A, 3C–D) and networks of salt bridges surrounding the catalytic site. In particular, in the proximity of the catalytic site, E88 and R526 are located, and they were demonstrated to be crucial for ApAAP activity by mutagenesis [35]. E88-R526 is also an invariant interdomain salt bridge in the POP family with relevant functional roles [50]–[52]. Our MD investigation shows that the salt bridge is involved in a more complex local network of salt bridge interactions, which includes, directly interacting with R526, also the catalytic aspartate D524 and concurs to maintain the correct orientation of D524 side chain in the catalytic site (Figure S3 and further details in Section 3.4). In fact, both sides of the catalytic triad are surrounded by salt bridge networks in cluster 1. These networks participate to the maintenance of the local architecture of the catalytic site. In particular, the E88-R526 mediated network (R113-E88-R526-D482-R113) is located on one side of the catalytic site, whereas, on the opposite side the D553-K85-D563-K566-D553 network and the K566-E562 salt bridge are located, connecting the interdomain region where the catalytic site resides with the α13 C-terminal helix (Figure 3C), which in turns faces the α1-helix. Cluster 1 also includes an extended salt bridge network which links, in proximity of the catalytic site, the β-propeller to the C-terminal domain composed by K110-E131-R486-E479-R497-E475-R501-D473 (Figure 3D).

Cluster 2, at which also α1 charged residues participate, includes the area between the two faces of the interconnected salt bridge belonging to cluster 1 (Figure 3B and 3E, Table S4). Clusters 3 and 4 are on opposite sites and include two different small clusters with few residues belonging to the N-terminal and the C-terminal domain, respectively (Figure 3B, Table S4).

#### Salt bridge networks in the surrounding of α1-helix can replace the interactions mediated by R18 and D15

The description of electrostatic interaction networks and their persistence, provided above, pointed out other interesting charged residues also in α1-helix region, which modulate protein dynamics and the interconnection between different structural elements. These residues have still not been experimentally investigated. In particular, focusing our attention on the proximity of α1-helix (Figure 3E), a pivotal role of E8 emerges in mediating several electrostatic interactions with α13 residues (E580-R581/R579), which in turns, by a chain of charge-charge interactions, connects this region to residues E438 and K463 on one side, and to the catalytic region K566-E562 (cluster 1) on the other side, as also discussed above. In the proximity of R18 and D15, the C-terminal residues D325 and R327 are the most important interconnected residues, which mediate, through a chain of electrostatic interactions, the communication from the α1-helix toward the rest of the N-terminal β-propeller domain (Figure 3C). Our dynamic framework enforces the notion that the lack of R18 and D15, which was previously investigated [32], can be overcome by other charged residues or in α1-helix (E8) or even in α1 surrounding (D325 and R327). Therefore, the mutations of R18 or D15 alone do not account for the effects induced upon α-helix deletion and do not affect protein activity and stability [32]. In fact, according to our MD framework, important salt bridge networks can still be formed in this region by residues as E8, D325 and R327.

#### Hydrophobic α1-helix residues provide long range transmitted effects to the catalytic site

It is nowadays well accepted that enzyme dynamics is intrinsically related to protein activity and stability, as well as residues with correlated motions are likely to be functionally correlated [7], [19]. Therefore, we calculated by dynamical cross-correlation matrix (DCCM) the most significant coupled motions in both wild type and ApAAP-Δ21 using different time windows (1–5–10 ns). Correlation cutoffs of 0.4, 0.45 and 0.5 have been tested to evaluate the pair of both positively and negatively correlated residues characterized by the most relevant averaged correlation values and a detailed analysis of the pairs of correlation and their spatial organization on the 3D structure have been carried out (Figure S3). In particular, only positively coupled residues have been identified. Interestingly, α1 does not feature over the different time windows a great number of directed correlated motions with other protein regions independently of the applied cutoffs for significant correlations (*data not shown)*.

**Figure 4:**
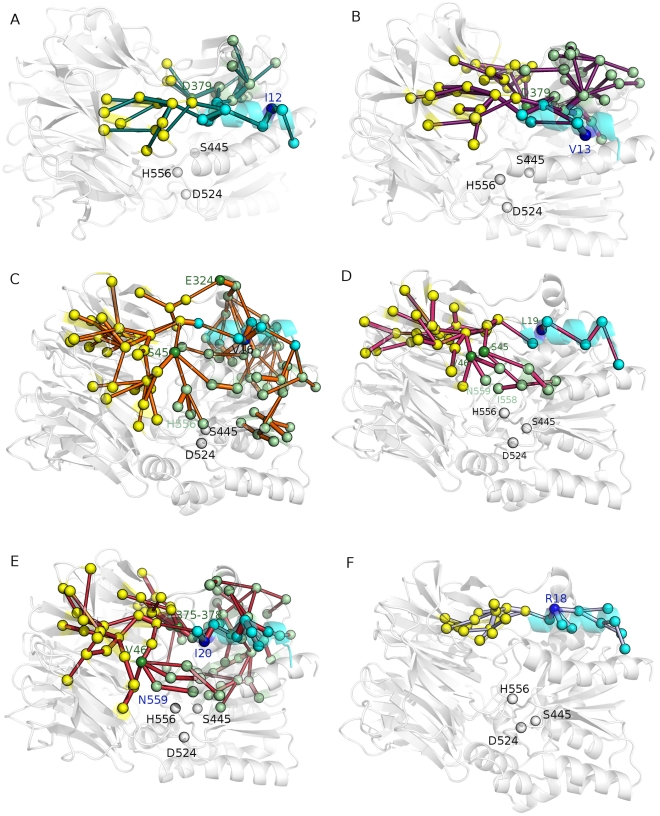
Chained correlations mediated by hydrophobic residues belonging to α1 helix. The chained correlations are indicated by marine (A), purple (B), orange (C), pink (D) and red (E), using I12 (A), V13 (B), V16 (C), L19 (D) and I20 (E) as root residues, respectively. The thickness of the sticks is proportional to the correlation values. Each residue belonging to the map of chained correlation is shown as a sphere. The root residue is colored in blue, whereas the residues belonging to the N-terminal α1-helix, the C-terminal and the N-terminal domains are colored in cyan, light green and yellow, respectively. Dark green spheres indicate central residues in the map of chained correlations which mediates communication to both the N-terminal and C-terminal domains. The catalytic triad is shown by spheres colored in light green if they belong to the chained correlations path or white if they are not included in the map. A correlation threshold of 0.35 in absolute value has been employed. The first depth and width threshold used in the calculations are of 5 and 4, respectively and the search is iterated for increasing depth value until no more correlations can be identified (more details in the Method section).

Only few residues of α1 emerge as characterized by locally coupled motions with their surroundings. Nevertheless, the *in silico* alanine scanning (Figure 1B) and the analysis of the intramolecular interaction networks (Figure 1C–D), as well as the results from PCA (Figure 2), point out effects which can be mediated by α1 hydrophobic residues. Therefore, to better clarify the role of the α1 hydrophobic residues, I12, V13, V16, L19 and I20 were used as root residues to calculate chained correlations (see Materials and Methods for details) in order to describe pathways of long range communications (Figure 4).

All the hydrophobic residues (I12, V13, V16, L19, I20) mediate interactions from the N-terminal helix to different regions of the β-propeller domain and the area of the C-terminal domain including the helices α-3, α-4, α-6, and the β-sheet (Figure 4A–E). On the contrary, only V16, L19 and I20 (Figure 4C–E) promote interactions which also bring to the catalytic site and the α13 helix, including in the case of V16 even the catalytic hystidine H556. Moreover, to enforce the relevance of the hydrophobic residues of α1-helix in mediating long range interactions and dynamics, the same analysis has been carried out using as a root residue R18 (Figure 4F), which if mutated to alanine does not affect protein stability or activity [32]. The chained correlations mediated by R18 are specifically localized in the residues of the N-terminal domain in the immediate proximity of the α1-helix and do not involve the C-terminal domain or distant region in the β-propeller domain.

Therefore, among all the α1-helix residues, V16, L19 and I20 are likely to account for the most relevant long range effects transmitted by the helix to secondary structural elements of both the N-terminal and C-terminal domains, but even directly to the catalytic site.

**Figure 5:**
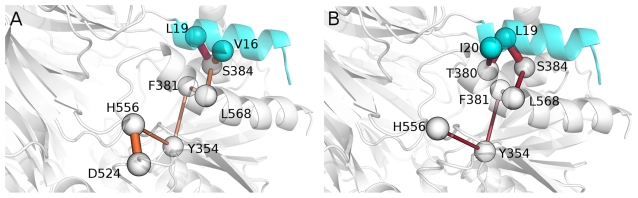
The shortest communication paths from the hydrophobic residues of the α1-helix to the catalytic site. The shortest and highest frequency pathways, as detected by PSN-DCCM analysis, between V16 (A), L19 (A), I20 (B) and the catalytic H556 and D524 are shown as sticks proportional to the intensity of the correlation.

In order to specifically identify the paths of communication to the catalytic site, a combined Protein Structure Network PSN/DCCM approach was also employed (see Materials and Methods for details). In fact, it aims at quantitatively defining the shortest paths of communication within the protein structure during dynamics between the hydrophobic residues of the α1-helix and the catalytic site (Figure 5). In line with the evidences collected so far, the algorithm fails to identify any significant communication for V13 and R18. In the case of I12, a short path is identified with a low and negligible frequency (<1.5%) among all the possible paths from this residue to the catalytic hystidine. Interestingly, for V16 and L19 the same main path is identified (with frequencies higher than 25%) that brings to both the catalytic hystidine and aspartate, and includes S384 -> L568 -> F381-> Y354 -> H556 -> D524 (Figure 5A). Also I20 communicates with the catalytic hystidine but along a slightly different path (I20->T380->L19->S384->L568->F381->Y454->H556) (Figure 5B). Interestingly, for each of the ApAAP mutants V16A, I19A, L20A the corresponding paths are lost and also the paths mediated by the not mutated residues are reduced in frequency (<2%), demonstrating the high relevance of these three hydrophobic residues in mediating the long range communication from the α1-helix to the catalytic site.

#### The lack of α1-helix and its mutations causes alteration in dynamic properties of the active site and coupled motions governing the catalytic triad

In light of the previous observations, also dynamics of the catalytic site of ApAAP-Δ21 and the ApAAP mutants have been investigated in details. In fact, ApAAP-Δ21 does not feature overall changes in secondary structure. Therefore, more subtle modifications in protein dynamics and interactions are expected. In principle, one might expected, in the absence of α1, a local solvent-exposition of hydrophobic patches. However, we did not identify, during dynamics, clear effects in this direction, probably due to a rearrangement of the α-helices of the C-terminal domain, in particular α13 and α4, which approach each other in the ApAAP-Δ21 simulations (*data not shown*).

**Figure 6:**
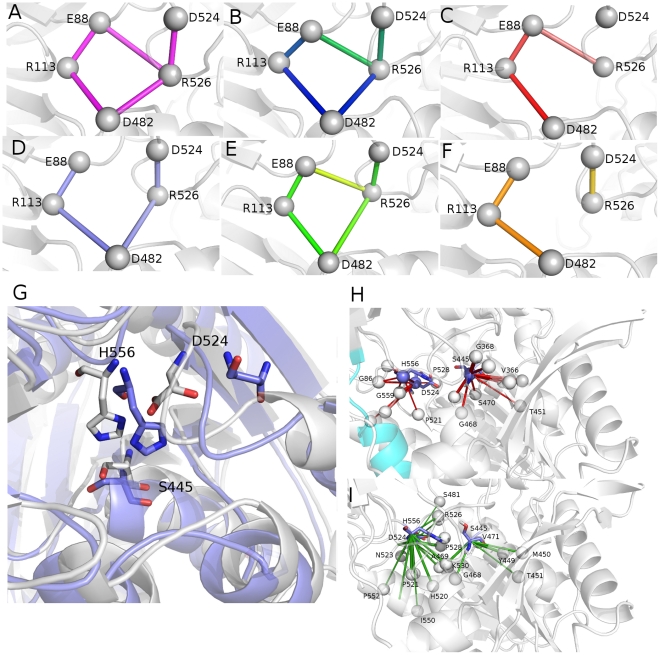
α1 deletion perturbs the architecture of ApAAP active site. A–F) Local network of salt bridge interactions mediated by R526 in the wild type ApAAP (A), ApAAP-Δ21 (B), ApAAP-I12A (C), ApAAP-V13A (D), ApAAP-V16A or ApAAP-I19 (E), ApAAP-L20A (F) are shown with different shade of color which are proportional to the persistence of the interaction during dynamics (with the darker colors indicating an higher persistence). G) wild type ApAAP and ApAAP-Δ21 average structures from the simulations are shown in white and blue, respectively. The catalytic residues are indicated by sticks. H–I) Coupled motions of the catalytic triad. The coupled motions which involve the catalytic triad are shown for wild type ApAAP (red sticks, H) and ApAAP-Δ21 (green sticks, I). Catalytic residues are shown as sticks and the α1-helix highlighted in cyan.

Otherwise, modifications in the electrostatic interaction networks and coupled motions can be detected, both in the ApAAP-Δ21 and the different ApAAP mutants. In particular, alterations can be observed in the surrounding of the catalytic site at expense of the catalytic D524 residue (Figure 6). In fact, the catalytic Asp in POP family is crucial to allow the correct plane orientation of the imidazole ring of the catalytic His and also to stabilize the ion pair interactions between the His and the negatively charged tetrahedral intermediate [28], [53]. Upon α1 deletion, an outward displacement of the catalytic D524 can be observed in the ApAAP-Δ21 simulation, which also causes a higher flexibility and displacement of H556, affecting the correct and functional architecture of the catalytic site (Figure 6G). These effects are also related to perturbation of the electrostatic interaction networks in the proximity of the catalytic site. In fact, D524 is maintained in the correct orientation inside the catalytic pocket by electrostatic interactions with R526 [28], [31], which is present with high persistence in the wild type ApAAP MD ensemble (Table S4). In wt ApAAP, R526 belongs to a well connected and balanced local network of charge-charge interactions, with very high persistence during dynamics (Figure 6A) including R526-E88, E88-R113, D482-R113 and R536-D482. Mutations of R526 or E88 were demonstrated to have detrimental effects on protein activity [35]. Moreover, mutations of the catalytic D524 compromising protein activity have been shown to cause a rearrangement in the surrounding residues [33]. According to our model, this can arise from the disruption of the aforementioned electrostatic network, bringing R526 to mainly interact with D482. In ApAAP-Δ21 simulations, the same network has been affected by the absence of the α1-helix (Figure 6B), probably due to the highest conformational freedom of D524 and H556 (Figure 6G). Therefore, the salt bridge between D524 with R526 is weakened, as well as the crucial salt bridge between R526 and E88, and they can be observed only in the first part of the ApAAP-Δ21 simulation. On the contrary, the interaction between R526 and D482 features an increased persistence and it is favored in ApAAP-Δ21 system. Similar effects are achieved in the ApAAP mutants, in which the electrostatic networks of interactions involving D524 are also modified (Figure 6D–F). In particular, V16A and L19A mutations show the same alteration in these local networks that can be highlighted for the whole α1-deletion (Figure 6B). The other ApAAP mutants feature even a decreased persistence (∼18%) of the E88-R526 interaction, as well as of R526-D524 below the significant cutoff of persistence.

In light of the evidences collected above, α1 deletion is likely to cause a perturbation in protein dynamics which is transmitted to the catalytic site. The deletion of this secondary structure element which is not in direct contact with the catalytic site causes detrimental effects on the catalytic site arrangement itself, implicating complex long range effects. Therefore, in order to provide a detailed description of protein dynamics of native ApAAP, we also employed DCCM to study the local networks of communication directly involving the catalytic residues. Coupled motions of ApAAP-Δ21 (Figure 6I) have been compared to the coupled motions of ApAAP wild type (Figure 6H). Wild type ApAAP presents two nuclei of precisely located correlated motions, which concur in the reciprocal orientation of the S445 side chain on one side of the catalytic site, and D524 and H556 on the other side (Figure 6H). Several glycine and proline residues contribute to these networks of coupled motions in the active site (Figure 6H). They are lost in the ApAAP-Δ21 simulations (Figure 6I). In fact, a remarkable difference in coupled motions of the catalytic triad in ApAAP-Δ21 is a tight network of positively correlated motions close to the catalytic site not present in the wild type enzyme. This may be a consequence of the conformational changes induced in the catalytic site of ApAAP-Δ21, affecting above all D524 and H556 (Figure 6I). Interestingly, similar alterations in the coupled motions around the catalytic site can be inferred from the V16A, L19A and I20A simulations (*data not shown*).

### Interdomain Interactions and Coupled Motions at the Interface between the Protein Domains Mediate the Conformational Changes to Achieve an ApAAP Open Conformation

**Figure 7:**
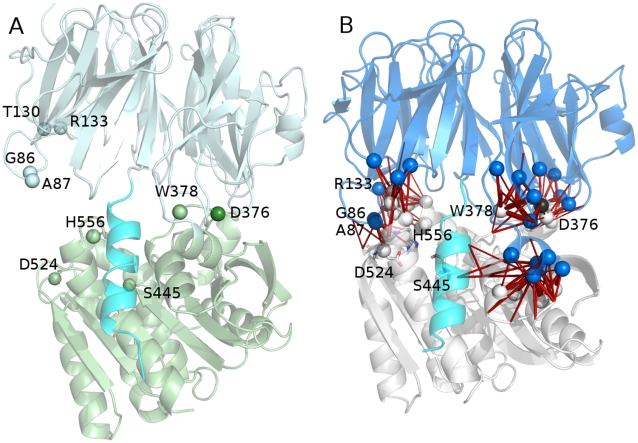
Correlated motions at the interdomain interface. A) The open structure of ApAAP identified by X-ray crystallography [33] is shown as a reference. The observed crucial residues for mediating cross-correlated motions in the simulations of the ApAAP closed form (panel B) are shown as spheres. B) The dynamical cross-correlations at the interdomain interface (correlation threshold of 0.4) in wild type ApAAP are shown as red lines. The β-propeller and the catalytic domains are shown in pale-cyan (A)/blue (B) and pale-green (A)/white (B), respectively, whereas the α1-helix is highlighted in cyan. The hinge residue proposed for the opening of the catalytic cleft, D376 is shown in dark green (A) and black (B), respectively.

As mentioned in the Introduction, a conformational selection mechanism has been recently proposed for ApAAP, according to which the enzyme can exist both in closed and open conformations (Figure 7A and 7B, respectively). The switch between these two conformational states is likely to be mediated by modification of intermolecular interactions between the two domains and relying on the hinge role of D376 [33]. A similar conformation has been also identified by electron microscopy investigation of a POP enzyme [54]. The open form can be the competent form for substrate recognition, whereas the closed conformation should be the catalytically active form, which ensures the correct reciprocal orientation of the catalytic triad [33]. In our simulations, a ns timescale is not able to detect conformational change of this entity but the analysis of persistence and intensity of the intramolecular interactions and cross-correlated residues could provide indication of possible hinge points for opening/closing of the structure and validate the model proposed by Polgár’s group [33].

**Figure 8:**
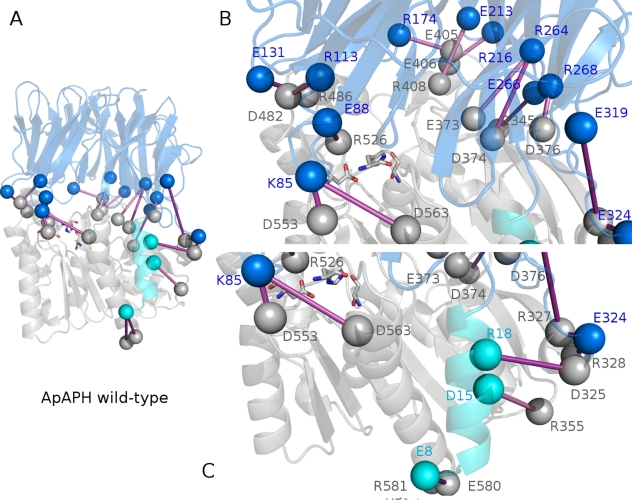
Salt bridges at the interdomain interface in wild type ApAAP. A general view (A) and zoom on the upper and lower regions (B, C) are shown. Residues involved in salt bridges and their networks are indicated as spheres connected by lines of different shade of magenta according to their persistence in the MD ensemble (from light to dark magenta for increasing persistence values). The β-propeller domain and the catalytic domain are highlighted in marine and white, respectively, whereas the α1-helix in cyan. Catalytic residues are shown as sticks.

**Figure 9:**
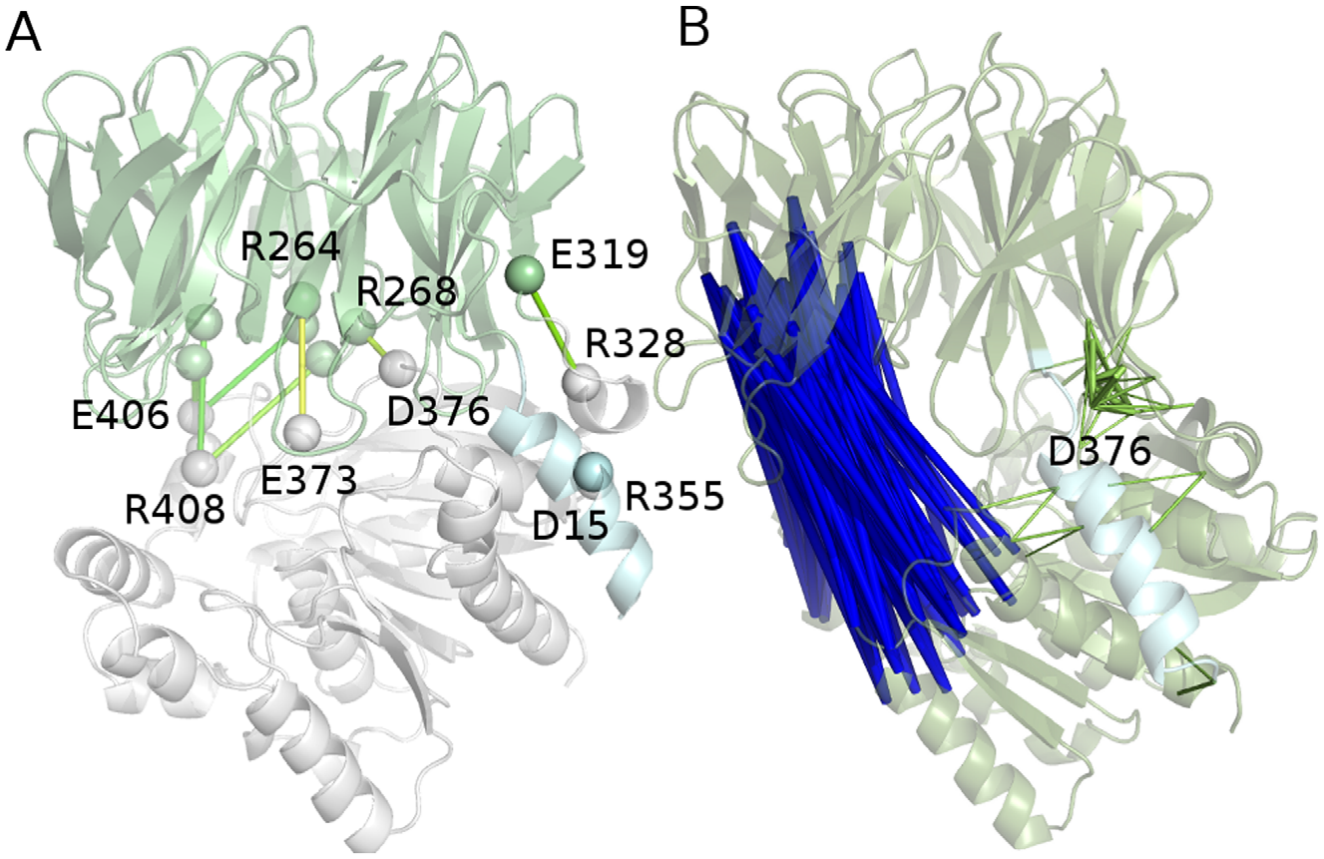
Simulations of ApAAP in open conformation. A) The cross-correlated motions at the interdomain interface (correlation threshold of 0.4) in simulations of open ApAAP are shown as green lines (positive correlations) and blue lines (negative correlations). The β-propeller and the catalytic domains are shown in pale-green and white, respectively, whereas the α1-helix is highlighted in pale-cyan. D376, which is the hinge residue proposed for the opening of the catalytic cleft [33] is shown. B) The salt bridge networks at the interface between the β-propeller and the catalytic domains in ApAAP open conformations are shown as spheres connected by yellow/green lines according to their persistence. The β-propeller domain and the catalytic domain are highlighted in pale-green and white, respectively, whereas the α1-helix in pale-cyan. Catalytic residues are shown as sticks.

In fact, D376, in our simulations starting from the closed conformation, is involved by interactions with R268 in one of the 18 salt bridges at the interface between the two ApAAP domains (Figure 8, Table S5). The interactions carried out by D376 are highlighted in the zoom of the interdomain interface shown in Figure 3B, where each pair of residues involved in a salt bridge is connected by a stick, colored according the persistence of the interaction in the MD ensemble. In the surrounding of D376 a tight network of salt bridges is located, characterized by high persistence and conservation in most of the simulation frames (E213-R408, R264-E373), including D376-R268 itself (Table S5, Figure 8B). These interactions are also conserved in simulations starting from the open ApAAP conformation (Figure 9, where the pairs of salt-bridges are indicated by sticks as in Figure 8 but with different shade of colors). On the contrary, other interface salt bridges feature a low/medium persistence, and several of them (K85-D553 and E131-R486 in particular) are located on the opposite side with respect to D376 (Figure 8B), which was proposed to be involved in the major conformational changes for the opening of the catalytic cleft (the open conformation is shown in Figure 7A as derived by the X-ray structure) [33]. The low persistence of these interactions in our MD ensemble is in agreement with the hypothesis of a region which can undergo toward conformational changes thanks to fluctuations around the native state [33]. In fact, these same salt bridges are not able to be established in the ApAAP open conformations, as also indicated by the simulations of the open ApAAP (Figure 9A). These results also are confirmed by the analysis of coupled correlated motions at the interface between the two protein domains (Figure 7B in the closed form). In fact, few coupled residue pairs and networks are identified at the interface between the two domains in the closed form (Figure 7B). In particular, a first and thick nucleus of correlated motions is localized on the side where D376 is placed, with D376 in a central position in the networks (Figure 7B). This group of interdomain correlations is also conserved in the simulations of ApAAP in the open conformation (Figure 9B). On the opposite side, weaker and fewer positively correlations (mainly involving G86, A87, R133 and D524 itself) have been identified in the closed ApAAP simulations (Figure 7B) and are absent in the open form.(Figure 9B). Indeed, this is the region characterized by the conformational changes that promote the opening of ApAAP.

Moreover, in the MD ensemble of the open ApAAP conformation, it is possible to identify, a group of dynamical anticorrelations (showed in blue in Figure 9B) between the residues of the N-terminal domain and the residues of the C-terminal domain (Figure 9B). These anticorrelated motions, which are located on the opposite site with respect to D476, indicate the tendency of the residues of the N-terminal domain to approach to the C-terminal domain, in order to restore a closed conformation. This can also be highlighted by the concomitant decrease of the protein radius of gyration in the simulation of open ApAAP (Figure S4), which is a clear indication of conformational changes promoting a closed an more compact form.

**Figure 10:**
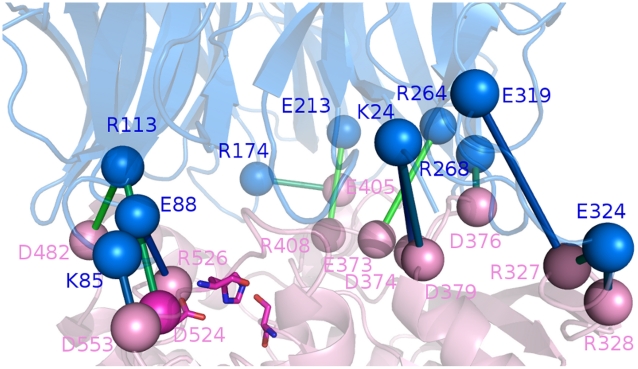
Salt bridge interactions at the interface between the two protein domains in ApAAP-Δ21. The salt bridge pairs are indicated by lines and the residues involved in the salt bridges and their networks as spheres. The catalytic residues are shown as sticks and the β-propeller and catalytic domain colored in marine and magenta, respectively. The salt bridges are connected by lines of different shade of colors according to their persistence in the MD ensemble (from green to blue for increasing persistence values).

The networks of salt bridges (Figure 10 and Table S5) and of correlated motions (data not shown) at the interface between the two domains are also affected by deletion of α1. In fact, this can be highlighted in the ApAAP-Δ21 simulation, in particular with the disappearance of K85-D563, E131-R486, R216-E406, R264-D374, and E266-R345 interactions (Figure 10). On the contrary, they are replaced by new interactions which also can affect, locally or long range, protein dynamics, as discussed above, as D374-K24-D379 ion pairs.

## Discussion

Intramolecular weak interactions are well known to play a crucial role in stabilizing protein structure, influencing protein dynamics and, in turn, function. The present study provides a detailed and comprehensive analysis of protein dynamics signature of a thermostable enzyme belonging to the POP family and the AAP family We identified several residues which can be suitable target for further biochemical studies and protein engineering both in the α1-helix (V16, L19 and I20) and at the interdomain interface (K85, G86, A87, E131, R133 and R486). In fact, it turns out a complex network of correlated residues and weak and low persistence electrostatic and hydrophobic interactions modulating the native state of AAP, as well as long range distal effects mediated by the α1-helix.

α1-helix has been shown important for thermal dependence of ApAAP catalytic activity [32]. From our investigation, α1-helix turns out to be a flexible structural element, residing on a complex network of electrostatic and hydrophobic interactions, which alone could not account for the whole effects induced by the α1-helix. The flexible nature of this region implies its ability to adapt to mutational events by rearranging salt bridges [32] and probably also varying hydrophobic interactions. This mechanism is also in agreement with similar previous mechanisms proposed for p53 structural stability, relying on the existence of a “fluid” salt bridge cluster [55], or for the HEAT repeats [56], where an unusual flexibility of hydrophobic interactions has been pointed out. In fact, in HEAT repeats a hydrophobic cluster of residues was demonstrated to be able to adapt to external changes by an internal rearrangement of its hydrophobic residues and their interactions. Moreover, cases in which protein regions characterized by charged residues in a hydrophobic environment, as the case of ApAAP α1, can destabilize the region and impart flexibility are known [57].

The emerging picture from our simulation data is that α1-helix is required to locally provide the correct orientation of several residues in the C-terminal regions, in particular in α13, α4 and α3 helices and long range to maintain a proper reciprocal orientation of the catalytic triad. The architecture of the surroundings of α1-helix and its long range effects mainly depend on its hydrophobic residues. Our study in particular points out a relevant role for V16, L19 and I20. The dynamical communication from α1-helix is transmitted, through α13, to the catalytic site and, through α3 and α4, to the β-propeller and other sites in the C-terminal domain.

ApAAP can provide a further example of enzyme in which thermal inactivation and thermal unfolding are decoupled, as pointed out in several and differently temperature adapted enzymes [43], [58]–[60]. In this scenario, changes in dynamics and intramolecular interaction networks and coupled residues, which are promoted by increasing temperatures, affect locally the active site architecture, but not the whole 3D structure and precede the onset of major thermal unfolding, with α1-helix being a structural element protective for this kind of events.

In details, the structural long range communication mediated from the N-terminal α1-helix to the catalytic site passes through the α13 helix and acts in particular on the catalytic hystidine and aspartate. If α1 is missing or his pivotal hydrophobic residues are mutated and no longer able to transmit this effects, protein dynamics of the catalytic site is affected and also the network of salt bridge interactions in the surrounding, transmitting the effects also to the POP invariant salt bridge E88-R526 at the domain interface. Moreover, the well defined and orchestrated coupled motions involving the catalytic triad are completely altered in the ApAAP-Δ21. This fact, along with the higher conformational freedom of H556 and D524 in the ApAAP-Δ21 and some of the most relevant alanine mutants, are in agreement with a low activity at ApAAP optimum temperatures in presence of the α1-deletion [32]. Moreover, in the same work ApAAP-Δ21 variant was demonstrated to be fully active at lower temperature with respect to the wild type protein [32], conferring to it a “mesophilic-like” behavior, as also suggested by Feng and coworkers [32]. These properties can be correlated to the higher flexibility in the proximity of the catalytic site which is a hallmark of several enzymes adapted to low temperatures [60], [61]. These results also well fit with the scenario provided for mesophilic-like mutations of a cold-adapted α-amylase [62]. The α-amylase mutants were shown not only able to restore the kinetic and thermodynamic properties of its warm-adapted counterpart [62] but also to restore the mesophilic dynamics fingerprint acting by long range effects on specific regions in the surrounding of the catalytic site [49]. Our data on dynamics of ApAAP and ApAAP-Δ21 enzyme, along with their experimental characterization [32], provide a second example of the possibility to modulate long range the protein dynamics signature of the catalytic site, acting on distal residues and at the same time to modify the thermal stability and catalytic activity of cold- and warm-adapted enzymes. They are two important examples which strongly encourage the application of protein dynamics and analysis of allosteric effects induced long range to the in silico prediction of sites for protein engineering.

At last, the results from our simulations, where also the wild type ApAAP open and closed conformations are compared, strongly confirm the mechanism proposed in the literature of a “clamshell-like” conformational rearrangement at the interdomain interface in the proximity of the catalytic site, recently proposed [33]. In this context, the study of correlated motions and the networks of electrostatic interactions at the interface in both open and closed ApAAP conformations on ns time scales, allows to disclose relevant interactions and couple motions which can contribute to the transition between the two forms and which deserve further attention. In fact, the atomistic resolution and the dynamic framework provided by classical MD allow to identify a subset of flexible and weakly connected residues (K85, G86, A87, E131, R486, D553) between the two domains which could trigger the conformational changes necessary to provide an AAP open conformation.

## Materials and Methods

### α-helices Definition

The secondary structures were assigned according to DSSP definition [63] and the numbering of the α-helices was assigned according to the definition provided in ref. [31] for ApAAP, including 13 α-helices numbered: α1 (10–21), α2 (239–243), α3 (324–329), α4 (380–387), α5 (405–409), α6 (418–432), α7 (446–457), α8 (474–480), α9 (483–493), α10 (497–502), α11 (505–511), α12 (529–541) and α13 (561–579). α1 and α2 belong to the β-propeller domain, whereas from α3 to α13 to the catalytic domain.

### *In silico* alanine Scanning

To assess the contribution of each residue of the N-terminal α1-helix to intramolecular protein stability, alanine mutants of ApAAP in the residues belonging to the α1-helix (residues 8–21) were considered, along with mutations in the surrounding residues (residues 22 and 23). The in silico alanine scanning calculations were carried out with 3 different methods on the X-ray structure of ApAAP (1VE6): FoldX 3.0 [64], I-Mutant 2.0 [65] and PoPMuSiC [66], which are based on different principles and implement diverse computational strategies. For PoPMuSic, the web-server version was employed.

The ΔΔG values are calculated from the unfolding Gibbs free energy value of the mutated protein minus the unfolding Gibbs free energy value of the wild type (Kcal/mol) both in FoldX and I-Mutant. PoPMuSiC indicates as destabilizing mutations those which totalize ΔΔG values >0 Kcal/mol, whereas I-Mutant and FoldX use as mark of mutations decreasing protein stability ΔΔG values <0 Kcal/mol. Therefore, only absolute values were employed for their comparison. In particular, a consensus of 2 among the 3 above mentioned methods was used to identify a subset of neutral (absolute ΔΔG values close to 0 Kcal/mol and not higher than 0.5 Kcal/mol), partial destabilizing (absolute ΔΔG values in the range of 0.5–1.2 Kcal/mol) or destabilizing (absolute ΔΔG values above 1.2 Kcal/mol) mutations.

### Molecular Dynamics (MD) Simulations

The X-ray structure of AAP the thermophilic archaeon Aeropyrum pernix K1 (ApAAP, pdb entry 1VE6 [31]), a truncated mutant of ApAAP that lacks the first short α-helix at the N-terminal (ApAAP-Δ21, pdb entry 2QZP [36]), along with wild type ApAAP in open conformation (pdb entry 3O4G [33]) and ApAAP I12A, V13A, V16A, I19A, L20A mutant variants (ApAAP-V16A, ApAAP-I19A, ApAAP-L20A) were employed as initial structures for simulations. MD simulations were performed using the GROMACS 3.3.3 software package (www.gromacs.org) implemented on a parallel architecture, using GROMOS96 43a1 force field.

The starting structures were soaked in a dodecahedral box of SPC (Simple Point Charge) water molecules [67] using periodic boundary conditions, with a minimum distance between the solute and the box of 0.7 nm. In order to neutralize the overall charge of the system, a number of water molecules equal to the protein net charge were replaced by Na^+^ ions.

The system was initially relaxed by molecular mechanics (steepest descent, 10.000 steps). The optimization step was followed by 50 ps of solvent equilibration at 283 K (time step 1 fs and thermal coupling constant of 1 fs), while restraining the protein atomic positions using an harmonic potential. The system was slowly driven to the target temperature (300 K) and pressure (1 bar) through a thermalization and a series of pressurization simulations of 50 ps each. The same preparation procedure was carried out for ApAAP-Δ21 and the ApAAP mutants.

Productive 100 ns MD simulations, depending on the system (Figure S1), were carried out in the isothermal-isobaric (NPT) ensemble, using an external bath with a coupling constant of 0.1 ps at 300 K. Pressure was kept constant (1 bar) by modifying the box dimensions and the time-constant for pressure coupling was set to 1 ps. The LINCS algorithm [68] was used to constrain heavy atoms bond lengths, allowing the use of a 2 fs time-step. Long-range electrostatic interactions were calculated using Particle-Mesh Ewald (PME) summation scheme [69]. Van der Waals and Coulomb interactions were truncated at 1.0 nm. The non-pair list was updated every 10 steps and conformations were stored every 4 ps. To identify recurring features and to avoid simulations artifacts two independent simulations (replicas) for the full-length ApAAP were carried out.

### Analysis of MD Simulations

The main chain root mean square deviation (rmsd), which is a crucial parameter to evaluate the stability of MD trajectories, was computed using the starting structure of the MD simulations as a reference and its time evolution was monitored. The first 5 ns of each simulations were discarded to ensure stability of the trajectories (Figure S1).

The analysis of the secondary structure (ss) content has been carried out using the DSSP program [63], along with the calculation of the most frequently attained secondary structure for each residue which was evaluated to obtain a residue-dependent persistence degree of secondary structure profile and to check stability of the secondary structure elements both in wild type ApAAP and mutant variants (*data not shown*).

### Dynamical Cross-correlation Matrices (DCCM)

Correlation plots were obtained by computing Cα dynamical cross-correlation matrix (DCCM) *C(i,j)* [18], using non over-lapping averaging windows of 1 ns, and also compared, for validation, to correlations on averaging windows of 5 and 10 ns. *C(i,j)* has been calculated according to
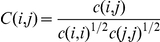
where *c(i,j)* is the covariance matrix element of protein fluctuation between residues *i* and *j*.

Only the most significant (|*C(i,j)*|>0.4) long range (|*i−j*|>12) positive and negative correlations were considered. In fact, the cutoff of distance in sequence was selected to exclude from the analysis the correlations relative to the α-helices or contiguous in the primary sequence. Moreover, since we discuss an average *C(i,j)* matrix, the cutoff of 0.4 (in absolute value) for significant correlation was selected to exclude from the analyses pairs of residues which are poorly communicating each other and likely to be characterized by uncoupled motions. To carefully verify that the analysis of an average *C(i,j)* matrix did not cause a loss of relevant information, the consistency between the average *C(i,j)* matrix with the individual matrices used in the averaging was evaluated. Correlations were then plotted on the 3D structures by connecting atoms *i* and *j* with lines, with thickness proportional to *C(i,j)*.

Chained correlations, using the residues belonging to α1-helix as root residues, were calculated for each system from post-processing of the DCCM of the atomic fluctuations. The chained correlations allow to highlight residues which are characterized by long range communication through the calculation of intermediate correlations. The same algorithm for chained correlations developed in FlexServ [70] was employed. According to this method, starting from a root residue the *w* highest correlated residues (*w* = width parameter for the search) are identified for *d* selected iterations (*d* = depth). We carried out chained correlations analysis with a *w* value equal to 4 and a threshold for most significant correlations in absolute value of 0.35. The correlation threshold was used to filter not correlated or weakly correlated residues. A first search was carried out for each root residue using values 4 and 5 for *w* and *d*, respectively. Then, the procedure was iterated for increasing *d* values since no more residues could be connected to the ones identified by the previous steps. The map of chained correlation was then filtered to identify the main path of long range communications, for each new node its connections with residues which bring to a “leaf node” and are not further connected within the map were discarded. Therefore, in the final only the connections for which the further nodes would “communicate” to a successive node were retained.

### Protein Structure Networks (PSN) and Shortest Correlated Path of Communication

The Protein Structure Network (PSN) approach [23] was integrated to data from the DCCM analysis of the wild type ApAAP simulation, to identify the most relevant communication pathways between residues of the α1-helix (I12, V13, V16, L19 and I20) and the catalytic triad (S445, D524 and H556), following an approach previously applied to other enzymes [71], [72]. The PSN method employs the graphs formalism to define a network of interacting residues in a given protein or protein complex from the number of non-covalently interacting atoms, using a calculated *I_ij_* interaction strength value as the edge weight, where *i* and *j* are residue identifiers. This value is calculated on the basis of the number of distinct atom pairs between residues *i* and *j* within a distance cutoff of 4.5 Å (n_ij_):
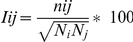
where *N_i_* and *N_j_* are normalization values for residues *i* and *j* obtained from a statistically significant protein dataset. Nodes are connected by edges when *I_ij_* > *I*_min_, where *I_min_* is a defined cutoff value. *I_min_* was set equal to *I_crit_*, where *I_crit_* is the value of *I_min_* at which the size of the largest clusters in the graph significantly changes (see ref. [23], [72]). To obtain a single PSN for each MD trajectory, a single PSN network was calculated for each frame and only edges present in at least half of the simulation frames were considered. For the selected residues (I12, V13, V16, L19, I20), the Floyd–Warshal algorithm was used to determine the shortest path between selected pairs of nodes in the PSN graph. The distance between connected residues was considered to be 1, and the shortest path was identified as the path in which the two residues were non-covalently connected by the smallest number of intermediate nodes. Only the shortest paths in which at least one identified node featured a significant correlation value (>0.35) with one of the residues of the selected pair were retained. All the PSN and PSN-DCCM calculations were performed using the WORDOM MD trajectories analysis suite [73].

### Principal Component Analysis and Root Mean Square Fluctuation

PCA reveals high-amplitude concerted motion in MD trajectories, through the eigenvectors of the mass-weighted covariance matrix (C) of the Cα atomic positional fluctuations [74]. In our trajectories (both wt, deletion and mutant ApAAP variants), the first three eigenvectors describe generally more than 40% of the total motion (in the range of 38%–43% of the total motion), and the first principal component accounts itself for about the 30% of the total variance.

The per-residue Cα root mean square fluctuation (rmsf) was calculated with respect to the average structure. To properly assess the flexibility profile, per-residue rmsf have been computed both on the whole trajectories, and also as average of per-residue rmsf profiles calculated on non-overlapping 10 ns time-windows. Moreover, to filter out noise due to non relevant fluctuations, rmsf has also been calculated on the trajectory filtered on the first 3 principal components, a well as a group of principal components accounting for the 70% of the total variance (which are in the range of 11 to 15 in the different ApAAP variants).

### Salt Bridges and Salt Bridge Networks

The electrostatic interactions were evaluated as oppositely charged groups at less than 0.45 nm of distance in at least 20% of the macro-trajectory frames. Also the angles between the charged groups involved in salt bridges were carefully checked before collecting the results. The persistence cutoff of 20% was selected as the persistence value that best divided the interaction dataset in well-separated groups, defined as signal and noise, according to a protocol previously applied [75] and also summarized in Figure S2. Moreover, in order to carefully verify the consistency of our analyses, the electrostatic cluster analysis was also carried out employing 0.4 and 0.5 nm of distance cutoffs, as well as taking into account salt bridges at lower persistences (*data not shown*). To identify clusters of salt bridge interactions, the residues involved in ion pairs have been represented as nodes of an unrooted unoriented graph, in which two nodes were connected by arcs if a salt bridge was identified between them or if they were at less than five residues of distance in the sequence. An exhaustive search procedure has been carried out on the graph to isolate spatial proximity clusters of electrostatic interactions.

### Hydrophobic Intramolecular Interactions During Dynamics

The hydrophobic interactions for the wild type ApAAP were calculated by PIC (Protein interaction calculator http://crick.mbu.iisc.ernet.in/~PIC/) server [76]. The hydrophobic interactions, carried out by the α1-helix residues, were calculated from the MD simulations to evaluate their persistence and stability in a dynamic framework. The surroundings of each of the N-terminal α1-helix residues, using a cutoff distance of 0.5 nm, were also monitored employing tools developed in our laboratory.

The interface intermolecular interactions between the two monomers of ApAAP dimer, which involve the α1 residues, was calculated by PROTORP [77] and PIC [76].

## Supporting Information

Figure S1 **Mainchain rmsd profiles over the simulation time of the different protein systems.** ‘r.’ indicates independent replicas of the same protein system, i.e. wild type ApAAP.
(PDF)

Figure S2 **Selection of significant cutoff for salt bridges persistence.** A salt bridge has been defined when two oppositely charged groups were found at less than 0.45 nm in at least one frame of the simulations. The persistence of each salt bridge interaction has therefore been calculated, in percentage, as the number of frames at which the salt bridge pair is identified divided by the number of total frames. Thus, distribution of the pairs at defined cutoff has been analyzed in terms of probability density function. It turns out that, in agreement with previous studied cases, there are several charged pairs at low persistence (<10%) and a second shoulder in the range of 10–20% of persistence, which are likely not to be relevant for protein structure and dynamics and identified as “noise” signal. Instead at persistence greater than 30%, the number of pairs at it is generally constant. The significant cutoff was set to 20%, since it best divides the dataset in two regions of low and high significance. This cutoff has been validated by adopting two supervised classification methods, trained with a set composed of two classes: noise, which comprises interactions below 10% of persistence, and signal, which comprised all the interactions over 30% of persistence. In particular, a Support Vector Machine (SVM) and a k-Nearest Neighbours (kNN, k = 4) classifier, as implemented in Matlab suite, have been trained on this set and used to classify all the interactions between 10 and 30%. The selected cutoff is indicated by a line.
(PDF)

Figure S3 **Correlation plot calculated from average DCCM on 1 ns (A–C) and 5 ns (D–F) timescales in wild type ApAAP.** Different cutoffs to selected correlations to plot on the 3D structure have been tested; 0.4 (A,D), 0.45 (B,E) and 0.5 (C,F). The β-propeller domain, catalytic domain and the α1-helix are colored in cyan, white and cyan respectively. Secondary structures are shown as cartoon and the catalytic triad as sticks.
(PDF)

Figure S4 **Gyration radius of ApAAP simulations starting from an open conformation,** in comparison to the simulations of closed ApAAP.
(PDF)

Table S1 **ΔΔG values of N-terminal α1-helix residues obtained comparing wild type and alanine mutant variants.** The **ΔΔ**G value of the residue **Δ**21 is not shown because it is already an alanine in the wild type ApAAP. The alanine mutations estimated to be destabilizing by I-Mutant and FoldX, are related to **ΔΔ**G lower than 0 Kcal/mol. On the contrary, in the case of PoPMuSiC the predicted destabilizing mutations are related to **ΔΔ**G higher than 0 Kcal/mol.
(DOC)

Table S2 **Contribution of α1 residues at the dimeric interface of ApAAP.** The intermolecular interactions involving side chains of α1 residues calculated by PIC on the X-ray structure (PDB entry 1VE6), along with the percentage of α1-helix area buried at the interface between the two monomers calculated by Protorp are shown. A and B indicate polypeptide chains A and B from the X-ray structure, respectively.
(DOC)

Table S3 **Hydrophobic interactions mediated by α1 residues and their persistence during dynamics.** ‘*’ Indicates interactions not present in the X-ray structure.
(DOC)

Table S4 **Salt bridge pairs and their persistence during the simulations.** On the left columns of the table salt bridges identified in the wild type ApAAP are reported, whereas on the right columns salt bridges identified only in the ApAAP-**Δ**21 are summarized. The main clusters of spatial proximity at which each salt bridge belongs are highlighted in blue (cluster 1), cyan (cluster 2), green (cluster 3) and yellow (cluster 4). In black, the persistence of the small and less significant populated clusters is shown.
(DOC)

Table S5 **Salt bridges pairs and their persistence localized at the interface between the two protein domains** for the wild type ApAAP and the ApAAP-Δ21. In bold interactions conserved in both the ApAAP variants are highlighted.
(DOC)
