# Supplementary figures and images for: Mechanisms of Intramolecular Communication in a Hyperthermophilic Acylaminoacyl Peptidase: A Molecular Dynamics Investigation
Source: PLoS One. 2012 Apr 27;7(4):e35686. doi: 10.1371/journal.pone.0035686 (PMC3338720; doi:10.1371/journal.pone.0035686)

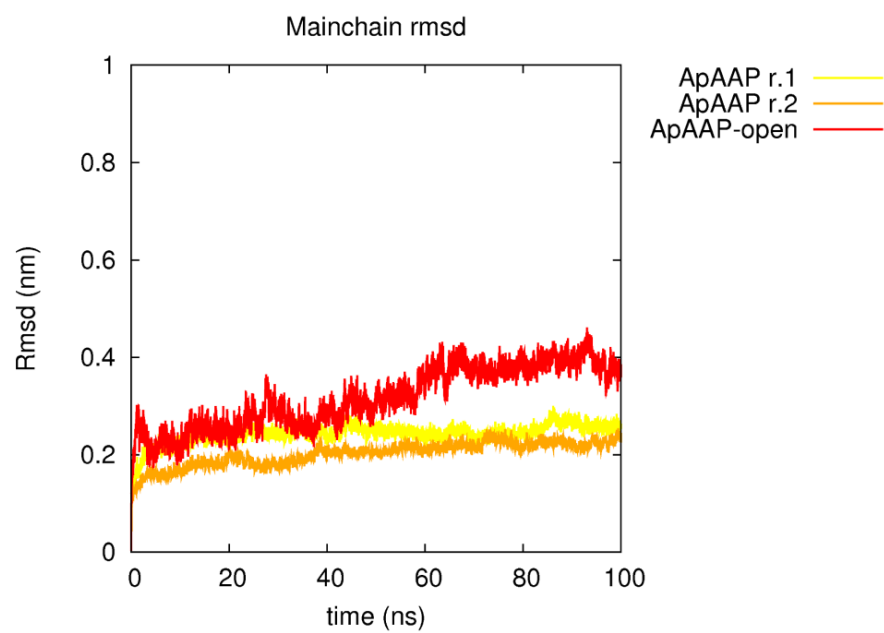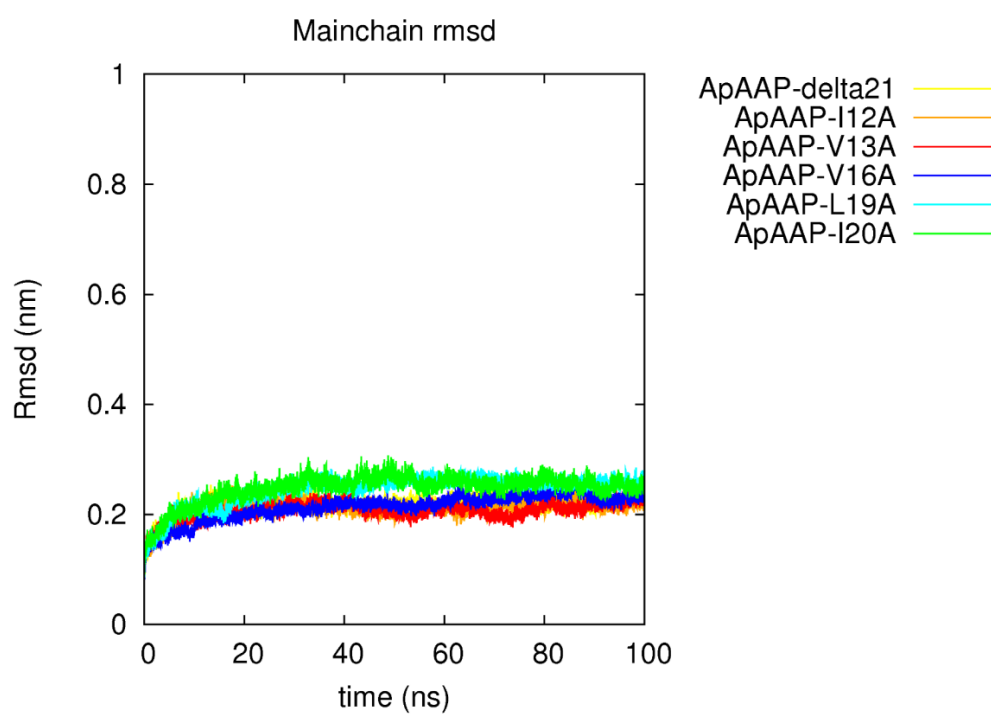

Supplement: Figure S1 — Mainchain rmsd profiles over the simulation time of the different protein systems. ‘r.’ indicates independent replicas of the same protein system, i.e. wild type ApAAP. (PDF) [file pone.0035686.s001.pdf]

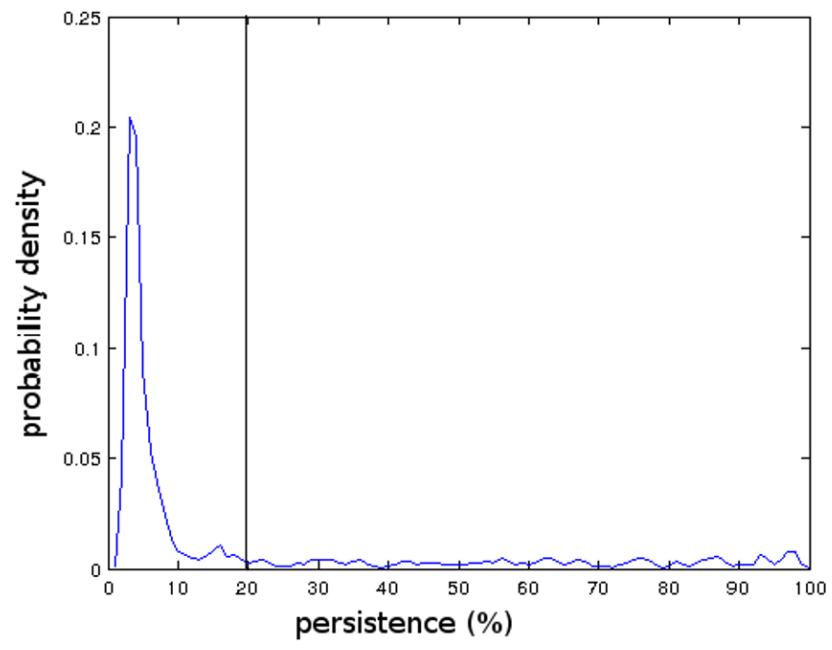

Supplement: Figure S2 — Selection of significant cutoff for salt bridges persistence. A salt bridge has been defined when two oppositely charged groups were found at less than 0.45 nm in at least one frame of the simulations. The persistence of each salt bridge interaction has therefore been calculated, in percentage, as the number of frames at which the salt bridge pair is identified divided by the number of total frames. Thus, distribution of the pairs at defined cutoff has been analyzed in terms of probability density function. It turns out that, in agreement with previous studied cases, there are several charged pairs at low persistence (<10%) and a second shoulder in the range of 10–20% of persistence, which are likely not to be relevant for protein structure and dynamics and identified as “noise” signal. Instead at persistence greater than 30%, the number of pairs at it is generally constant. The significant cutoff was set to 20%, since it best divides the dataset in two regions of low and high significance. This cutoff has been validated by adopting two supervised classification methods, trained with a set composed of two classes: noise, which comprises interactions below 10% of persistence, and signal, which comprised all the interactions over 30% of persistence. In particular, a Support Vector Machine (SVM) and a k-Nearest Neighbours (kNN, k = 4) classifier, as implemented in Matlab suite, have been trained on this set and used to classify all the interactions between 10 and 30%. The selected cutoff is indicated by a line. (PDF) [file pone.0035686.s002.pdf]

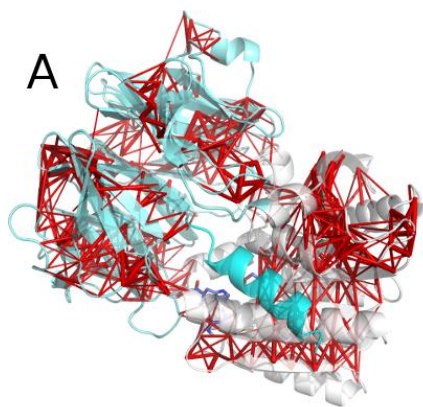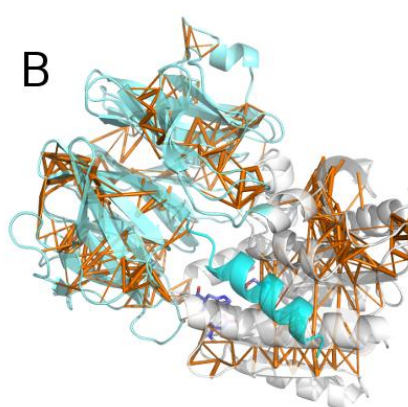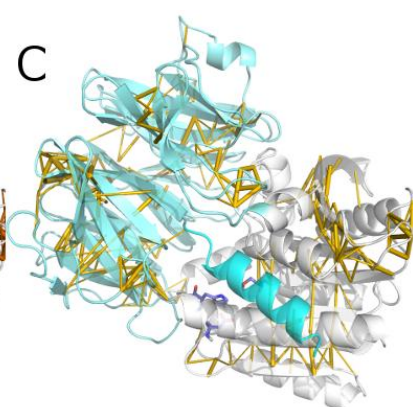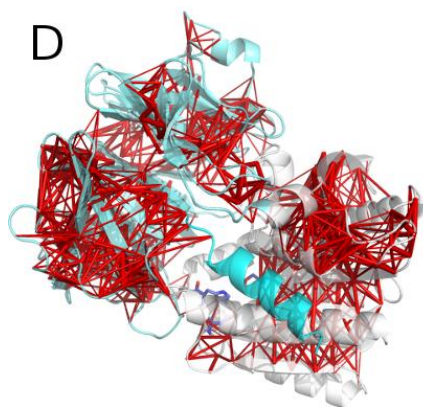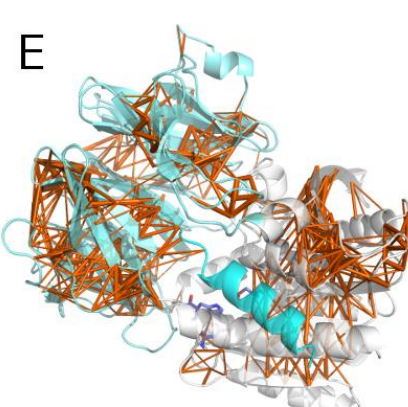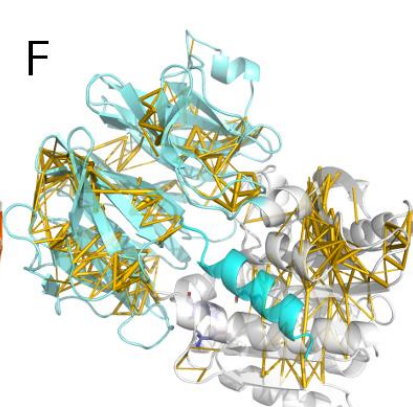

Supplement: Figure S3 — Correlation plot calculated from average DCCM on 1 ns (A–C) and 5 ns (D–F) timescales in wild type ApAAP. Different cutoffs to selected correlations to plot on the 3D structure have been tested; 0.4 (A,D), 0.45 (B,E) and 0.5 (C,F). The β-propeller domain, catalytic domain and the α1-helix are colored in cyan, white and cyan respectively. Secondary structures are shown as cartoon and the catalytic triad as sticks. (PDF) [file pone.0035686.s003.pdf]

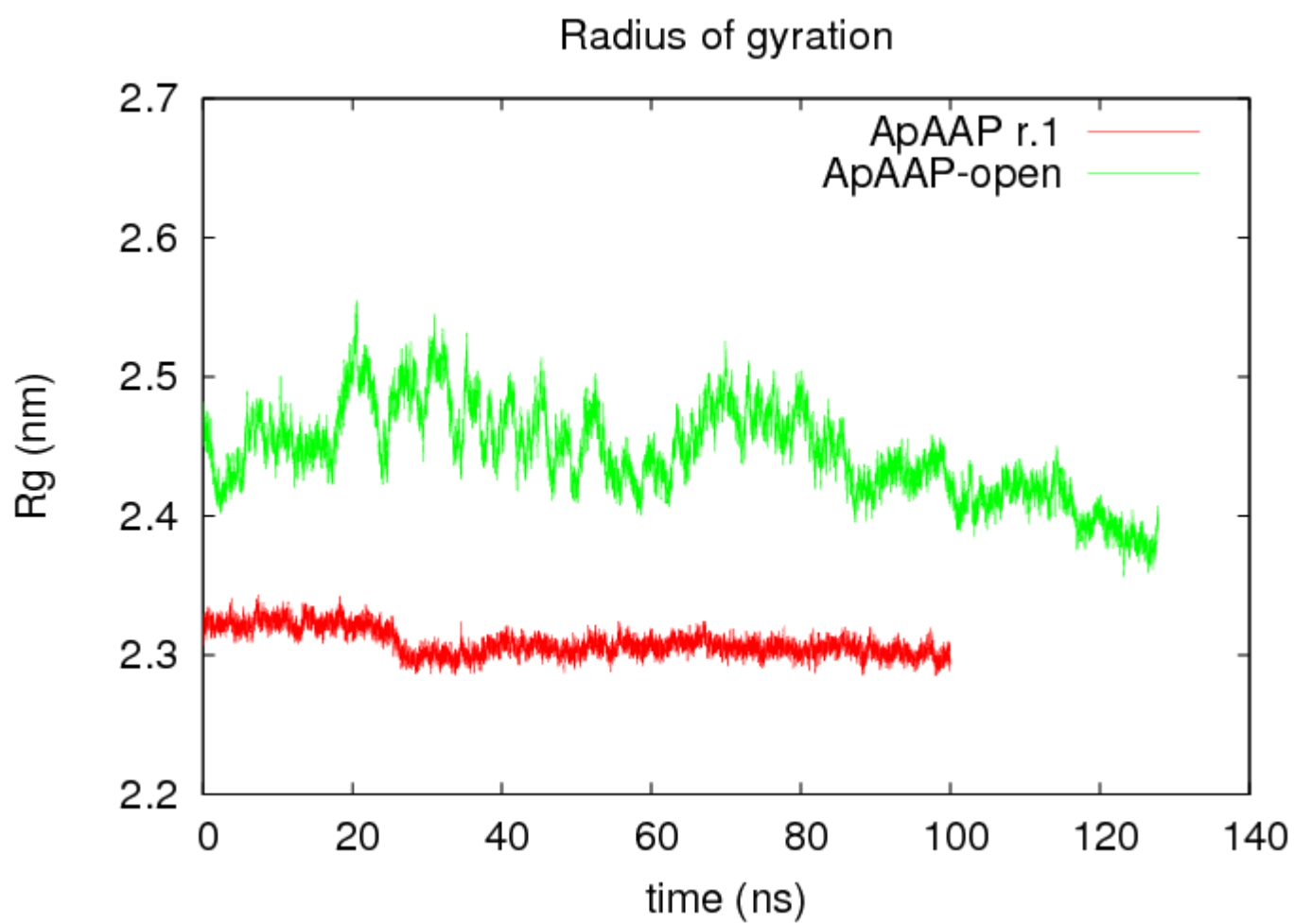

Supplement: Figure S4 — Gyration radius of ApAAP simulations starting from an open conformation, in comparison to the simulations of closed ApAAP. (PDF) [file pone.0035686.s004.pdf]
