# Supplementary material for: Mechanisms of Intramolecular Communication in a Hyperthermophilic Acylaminoacyl Peptidase: A Molecular Dynamics Investigation
Source: PLoS One. 2012 Apr 27;7(4):e35686. doi: 10.1371/journal.pone.0035686 (PMC3338720; doi:10.1371/journal.pone.0035686)
Supplement: Table S1 — ΔΔG values of N-terminal α1-helix residues obtained comparing wild type and alanine mutant variants. The ΔΔG value of the residue Δ21 is not shown because it is already an alanine in the wild type ApAAP. The alanine mutations estimated to be destabilizing by I-Mutant and FoldX, are related to ΔΔG lower than 0 Kcal/mol. On the contrary, in the case of PoPMuSiC the predicted destabilizing mutations are related to ΔΔG higher than 0 Kcal/mol. (DOC) [file pone.0035686.s005.doc]

|  | **I-mutant** | **FoldX** | **PoPMuSiC** |
| --- | --- | --- | --- |
| Residue | ∆∆G Kcal/mol | ∆∆G Kcal/mol | ∆∆G Kcal/mol |
| E8A | 0,03 | **-0,98** | -0,16 |
| F9A | **-1,08** | **-5,51** | 0,47 |
| S10A | -0,06 | **-3,17** | -0,35 |
| R11A | -0,68 | **-3,66** | 0,23 |
| I12A | **-2,35** | **-1,93** | **2,27** |
| V13A | **-1,68** | **-1,45** | **1,47** |
| R14A | **-1,13** | -0,45 | 0,62 |
| D15A | **-1,54** | **-5,29** | 0,45 |
| V16A | **-1,7** | **-2,49** | **2,63** |
| E17A | **-0,85** | -0,62 | 0,02 |
| R18A | **-1,67** | **-0,97** | **1,19** |
| L19A | **-2,93** | **-1,69** | **2,87** |
| I20A | **-3,26** | **-1,71** | **1,71** |
| V22A | **-1,54** | **-2,34** | **1,36** |
| E23A | **-0,71** | **-0,71** | 0,1 |
| K24A | **-1,31** | **-0,95** | **1,27** |
|  |  |  |  |
|  | ∆∆G < 0 destabilizing | ∆∆G < 0 destabilizing | ∆∆G > 0 destabilizing |
