# Supplementary material for: Mechanisms of Intramolecular Communication in a Hyperthermophilic Acylaminoacyl Peptidase: A Molecular Dynamics Investigation
Source: PLoS One. 2012 Apr 27;7(4):e35686. doi: 10.1371/journal.pone.0035686 (PMC3338720; doi:10.1371/journal.pone.0035686)
Supplement: Table S2 — Contribution of α1 residues at the dimeric interface of ApAAP. The intermolecular interactions involving side chains of α1 residues calculated by PIC on the X-ray structure (PDB entry 1VE6), along with the percentage of α1-helix area buried at the interface between the two monomers calculated by Protorp are shown. A and B indicate polypeptide chains A and B from the X-ray structure, respectively. (DOC) [file pone.0035686.s006.doc]

| **Residues** | **Protein-Protein Interactions** |
| --- | --- |
| 13V_A : 13V_B | Protein-Protein Hydrophobic Interactions |
| 13V_A : 573F_B | Protein-Protein Hydrophobic Interactions |
| 13V_A : 9F_B | Protein-Protein Hydrophobic Interactions |
|  |  |
|  |  |
| 8E_A : 562E_B | Protein-Protein Main Chain-Side Chain Hydrogen Bonds |
| 9F_A : 17E_B | Protein-Protein Main Chain-Side Chain Hydrogen Bonds |
| 9F_B : 17E_A | Protein-Protein Main Chain-Side Chain Hydrogen Bonds |
| 10S_B : 17E_A | Protein-Protein Main Chain-Side Chain Hydrogen Bonds |
| 17E_B : 8E_A | Protein-Protein Main Chain-Side Chain Hydrogen Bonds |
|  |  |
| 10S_B : 17E_A | Protein-Protein Side Chain-Side Chain Hydrogen Bonds |
|  |  |
|  | **% Interface ASA** |
| 8E_A | 9,04 |
| 10S_A | 4,64 |
| 13V_A | 2,87 |
| 17E_A | 4,32 |
