# Supplementary material for: Mechanisms of Intramolecular Communication in a Hyperthermophilic Acylaminoacyl Peptidase: A Molecular Dynamics Investigation
Source: PLoS One. 2012 Apr 27;7(4):e35686. doi: 10.1371/journal.pone.0035686 (PMC3338720; doi:10.1371/journal.pone.0035686)
Supplement: Table S3 — Hydrophobic interactions mediated by α1 residues and their persistence during dynamics. ‘*’ Indicates interactions not present in the X-ray structure. (DOC) [file pone.0035686.s007.doc]

| **Residues** | **Persistence of interaction (%)** |
| --- | --- |
| 9PHE-12ILE * | 99.20 |
| 9PHE-13VAL | 99.86 |
| 9PHE-573PHE | 12.77 |
| 9PHE-576ALA | 13.16 |
| 12ILE-13VAL * | 100.00 |
| 12ILE-16VAL * | 89.24 |
| 12ILE-387ALA | 30.18 |
| 12ILE-388ALA * | 69.06 |
| 13VAL-16VAL | 89.96 |
| 13VAL-569LEU* | 24.51 |
| 13VAL-573PHE* | 84.84 |
| 16VAL-19LEU | 99.98 |
| 16VAL-20ILE* | 96.49 |
| 16VAL-569LEU* | 37.63 |
| 16VAL-572VAL* | 13.16 |
| 16VAL-573PHE | 14.00 |
| 19LEU-20ILE | 100.00 |
| 19LEU-21ALA | 92.58 |
| 19LEU-326LEU* | 34.54 |
| 19LEU-378TRP* | 38.31 |
| 19LEU-383ALA* | 36.12 |
| 20ILE-22VAL | 89.69 |
| 20ILE-41PHE* | 57.04 |
| 20ILE-561MET* | 22.71 |
| 20ILE-564ALA* | 35.67 |
| 20ILE-568LEU* | 24.51 |
| 21ALA-22VAL | 100.00 |
| 21ALA-561MET* | 32.84 |
| 21ALA-41PHE | 60.29 |
| 22VAL-41PHE | 70.39 |
| 22VAL-378TRP | 73.57 |
