# Supplementary material for: Mechanisms of Intramolecular Communication in a Hyperthermophilic Acylaminoacyl Peptidase: A Molecular Dynamics Investigation
Source: PLoS One. 2012 Apr 27;7(4):e35686. doi: 10.1371/journal.pone.0035686 (PMC3338720; doi:10.1371/journal.pone.0035686)
Supplement: Table S4 — Salt bridge pairs and their persistence during the simulations. On the left columns of the table salt bridges identified in the wild type ApAAP are reported, whereas on the right columns salt bridges identified only in the ApAAP-Δ21 are summarized. The main clusters of spatial proximity at which each salt bridge belongs are highlighted in blue (cluster 1), cyan (cluster 2), green (cluster 3) and yellow (cluster 4). In black, the persistence of the small and less significant populated clusters is shown. (DOC) [file pone.0035686.s008.doc]

| **Salt bridge pairs** | **Macro-Trajectory wt** | **∆21** | **Salt bridge pairs** | **∆21** |
| --- | --- | --- | --- | --- |
|  |  |  | VAL22:GLU23 | 42.79 |
| GLU8:GLU580 | 26.35 | - | VAL22:GLU43 | 85.45 |
| GLU8:ARG581 | 24.93 | - | LYS24:ASP374 | 22.86 |
| GLU8:ARG11 | 38.26 | - | LYS24:ASP379 | 57.90. |
| ARG14:GLU17 | 32.42 | - | ARG113:GLU131 | 52.58 |
| ASP15:ARG18 | 35.53 | - | ARG81:ASP563 | 48.68 |
| ASP15:ARG355 | 95.61 | - |  |  |
| ARG18:ASP325 | 24.39 | - | ASP342:LYS410 | 79.62 |
| ASP32:LYS35 | 94.48 | 92.15 | ARG398:GLU406 | 28.11 |
| ASP34:LYS35 | 23.86 | 30.28 | LYS463:ARG581 | 27.65 |
| ASP34:ARG292 | 95.47 | 82.03 | ASP473:ARG534 | 41.14 |
| LYS35:ASP52 | 81.67 | 64.87 | GLU476:LYS530 | 21.43 |
| ASP52:ARG292 | 81.77 | 52.02 |  |  |
| ARG61:GLU102 | 40.30 | 67.00 |  | |
| ARG61:GLU103 | 47.51 | 30.04 |  | |
| GLU62:ARG81 | 70.23 | 73.38 |  | |
| ASP69:ARG287 | 96.75 | 98.52 |  | |
| ARG76:ASP119 | 89.36 | 26.73 |  | |
| ARG76:GLU122 | 38.80 | 25.06 |  | |
| ARG81:GLU103 | 73.69 | 22.43 |  | |
| LYS85:ASP553 | 31.68 | 70.60 |  | |
| LYS85:ASP563 | 50.74 | - |  | |
| GLU88:ARG113 | 96.43 | 66.32 |  | |
| GLU88:ARG526 | 55.69 | 60.16 |  | |
| ARG99:GLU102 | 41.98 | 26.11 |  | |
| ARG105:GLU107 | 30.71 | 30.81 |  | |
| LYS110:GLU131 | 39.36 | - |  | |
| ARG113:ASP482 | 98.75 | 90.08 |  | |
| GLU131:ARG486 | 32.13 | - |  | |
| ASP132:ARG133 | 56.47 | 24.27 |  | |
| ASP132:ARG149 | 44.42 | 60.01 |  | |
| ASP140:ARG145 | 50.38 | 68.76 |  | |
| GLU146:ARG149 | 59.95 | 72.89 |  | |
| ASP158:ARG160 | 99.33 | 97.23 |  | |
| ARG174:GLU405 | 71.23 | 22.23 |  | |
| GLU194:ARG219 | 23.53 | 56.54 |  | |
| LYS207:ASP224 | 56.17 | 52.43 |  | |
| LYS207:GLU231 | 40.28 | 59.24 |  | |
| GLU213:ARG408 | 95.70 | 99.99 |  | |
| ARG216:GLU406 | 73.51 | - |  | |
| GLU217:ARG219 | 58.04 | 33.18 |  | |
| GLU217:ARG244 | 51.30 | 73.36 |  | |
| ARG219:ASP232 | 58.16 | 59.90 |  | |
| ASP224:ARG226 | 39.24 | 32.42 |  | |
| GLU234:ARG244 | 50.37 | 49.93 |  | |
| ASP239:ARG277 | 93.13 | 25.79 |  | |
| ASP256:ARG258 | 56.95 | 30.92 |  | |
| ARG258:ASP274 | 30.85 | 26.29 |  | |
| ARG264:GLU373 | 91.85 | 99.28 |  | |
| ARG264:ASP374 | 43.02 | - |  | |
| ARG265:GLU266 | 79.26 | 56.14 |  | |
| GLU266:ARG345 | 28.67 | 24.60 |  | |
| ARG268:ASP376 | 67.32 | 55.32 |  | |
| ARG277:GLU279 | 28.15 | 37.96 |  | |
| LYS294:GLU315 | 57.40 | 57.44 |  | |
| ARG307:GLU319 | 61.43 | 75.35 |  | |
| GLU319:ARG327 | 23.04 | 42.60 |  | |
| GLU324:ARG327 | 43.46 | 54.07 |  | |
| GLU324:ARG328 | 44.02 | 45.41 |  | |
| ASP325:ARG328 | 35.80 | 21.50 |  | |
| ASP325:ARG355 | 42.35 | - |  | |
| ARG334:GLU352 | 89.19 | 47.47 |  | |
| GLU339:ARG345 | 54.84 | 69.96 |  | |
| ASP342:ARG398 | 50.59 | 35.57 |  | |
| ARG398:ASP422 | 95.46 | 90.92 |  | |
| GLU406:LYS410 | 34.74 | - |  | |
| LYS410:GLU419 | 37.88 | - |  | |
| ASP414:ARG503 | 86.16 | 89.20 |  | |
| GLU421:ARG428 | 32.32 | 20.14 |  | |
| GLU421:LYS458 | 44.78 | 59.96 |  | |
| ARG428:GLU432 | 30.64 | 41.29 |  | |
| GLU438:LYS463 | 90.85 | 82.75 |  | |
| GLU438:ARG579 | 38.91 | 53.60 |  | |
| ASP473:ARG501 | 26.45 | - |  | |
| GLU475:ARG497 | 71.60 | 89.56 |  | |
| GLU475:ARG501 | 69.03 | 94.60 |  | |
| GLU479:ARG486 | 73.01 | 51.56 |  | |
| GLU479:ARG497 | 32.11 | 71.50 |  | |
| ASP482:ARG526 | 63.43 | 97.27 |  | |
| GLU498:ARG501 | 35.90 | 38.11 |  | |
| ASP510:ARG511 | 25.25 | 71.35 |  | |
| ASP510:ARG542 | 60.69 | 57.87 |  | |
| ASP510:LYS544 | 28.48 | - |  | |
| ASP524:ARG526 | 77.33 | 28.00 |  | |
| ARG534:GLU538 | 33.64 | 26.01 |  | |
| GLU538:ARG542 | 78.43 | 89.35 |  | |
| ASP553:LYS566 | 47.23 | 43.09 |  | |
| GLU562:LYS566 | 52.44 | 76.44 |  | |
| ASP563:LYS566 | 43.52 | 78.68 |  | |
| ARG579:GLU580 | 55.30 | 35.67 |  | |
