# Supplementary material for: Mechanisms of Intramolecular Communication in a Hyperthermophilic Acylaminoacyl Peptidase: A Molecular Dynamics Investigation
Source: PLoS One. 2012 Apr 27;7(4):e35686. doi: 10.1371/journal.pone.0035686 (PMC3338720; doi:10.1371/journal.pone.0035686)
Supplement: Table S5 — Salt bridges pairs and their persistence localized at the interface between the two protein domains for the wild type ApAAP and the ApAAP-Δ21. In bold interactions conserved in both the ApAAP variants are highlighted. (DOC) [file pone.0035686.s009.doc]

| **ApAAP wt** | **Persistence (%)** | **ApAAP-∆21** | **Persistence (%)** |
| --- | --- | --- | --- |
| GLU8:GLU560 | 23.13 | LYS24:ASP374 | 22.86 |
| GLU8:ARG581 | 34.81 | LYS24:ASP379 | 57.90 |
| ASP15:ARG355 | 97.31 | ARG81:ASP563 | 48.68 |
| ARG18:ASP325 | 19.00 | **LYS85:ASP553** | 70.60 |
| **LYS85:ASP553** | 24.76 | **GLU88:ARG526** | 60.16 |
| LYS85:ASP563 | 53.97 | **ARG113:ASP482** | 96.07 |
| **GLU88:ARG526** | 53.26 | **ARG174:GLU405** | 72.97 |
| **ARG113:ASP482** | 98.54 | **GLU213:ARG408** | 99.99 |
| GLU131:ARG486 | 34.06 | **ARG264:GLU373** | 99.28 |
| **ARG174:GLU405** | 77.17 | **GLU266:ARG345** | 24.60 |
| **GLU213:ARG408** | 88.90 | **ARG268:ASP376** | 55.32 |
| ARG216:GLU406 | 70.25 | LYS294:GLU315 | 57.44 |
| **ARG264:GLU373** | 91.84 |  |  |
| ARG264:ASP374 | 41.23 |  |  |
| **GLU266:ARG345** | **28.07** |  |  |
| **ARG268:ASP376** | 67.20 |  |  |
| GLU319:ARG327 | 23.04 |  |  |
| GLU324:ARG355 | 40.81 |  |  |
